# Association of *FMR1* Genotypes with In Vitro Fertilization (IVF) Outcomes Based on Ethnicity/Race

**DOI:** 10.1371/journal.pone.0018781

**Published:** 2011-04-15

**Authors:** Norbert Gleicher, Andrea Weghofer, Irene H. Lee, David H. Barad

**Affiliations:** 1 Center for Human Reproduction (CHR) and Foundation for Reproductive Medicine, New York, New York, United States of America; 2 Department of Obstetrics, Gynecology and Reproductive Sciences, Yale University School of Medicine, New Haven, Connecticut, United States of America; 3 Department of Gynecological Endocrinology and Reproductive Medicine, Vienna University School of Medicine, Vienna, Austria; 4 Department of Epidemiology and Social Medicine and Department of Obstetrics, Gynecology and Women's Health, Albert Einstein College of Medicine, Bronx, New York, United States of America; Florida International University, United States of America

## Abstract

The *FMR1* gene, mapping to an area of the X chromosome closely associated with autoimmunity also affects ovarian reserve, with specific genotypes associated with distinct ovarian aging patterns. They, therefore, could also be associated with differences of in vitro fertilization (IVF) outcomes, reported between races/ethnicities. We analyzed 339 consecutive IVF patients, 232 Caucasian, 59 African and 48 Asian, for *FMR1* genotypes, and tested by multiple logistic regressions for associations between race/ethnicity, *FMR1* genotype, autoimmunity and pregnancy chances with IVF. *FMR1* genotypes were predictive of pregnancy (P = 0.046), *het-norm/low* most significantly and with decreasing chance in comparison to *norm* genotypes (OR 0.44; 95% CI 0.23–0.85; P = 0.014). Race/ethnicity was, overall, independently associated (P = 0.03), African demonstrating decreased odds in comparison to Caucasian (OR 0.33. 95%CI 0.13–0.79; P = 0.014). Autoimmunity did not differ but interaction of autoimmunity with *FMR1* genotype almost reached significance (P = 0.07). Logistic regression with race/ethnicity and interaction between *FMR1* genotype and autoimmunity in the model, demonstrated 2.5-times the odds of being associated with autoimmune positivity (OR 2.5, 1.34–4.55; P = 0.004). *FMR1* genotypes offer a possible explanation for differences in IVF outcomes between races/ethnicities.

## Introduction

Infertility treatment outcomes vary in different races/ethnicities [1]–[9], and disparities increase with improving outcomes [10]. This does not surprise: For example, primary ovarian insufficiency (POI, also called premature ovarian failure, POF) varies [11], menopause differs [12] and Chinese oocytes donors demonstrate more premature ovarian aging (POA, also called occult primary ovarian insufficiency, OPOI) than Caucasians [13] and higher estradiol levels [9]. Asians also present with milder polycystic ovary (PCO) phenotype than Caucasians and Africans [14], and African women in general experience excessive infertility [15]. Two studies demonstrated IVF cycle differences, though no difference in pregnancy rates/live births [9], [16]. Why outcome differences with intrauterine inseminations [5] or in vitro fertilization (IVF) [3], [4], [6]–[9] are observed, is unknown.

Distribution of fragile X mental retardation (*FMR1*) genotypes varies between Caucasian, African and Asian women [17]. The gene maps to the 5′ untranslated exon 1 on the X chromosome (Xq27.3) [18], and is primarily assessed in women attempting conception. Genotype classes, defined by length of polymorphic expansions of CGG nucleotides, denote increased risk towards mostly neuro-psychiatric conditions [19]. *FMR1* is one amongst a number of genes generating diseases via trinucleotide repeat expansions. Others are, for example, myotonic dystrophy and Huntington's disease [20].

At so-called premutation range genotype (American College of Medical Genetics, 55 to approximately 200 CGGs), and full mutation (above approximately 200–230), *FMR1* induces severe neuro-psychiatric conditions [in male premutation carriers fragile X – associated tremor/ataxia syndrome (FXTAS) a neurodegenerative disease]. Full mutations (fragile X syndrome) are the most frequent known genetic cause of autism and mental retardation in males. Because of X chromosome inactivation women are less and/or more mildly affected than males [19].

Women with premutations express the only *non*-neuro-psychiatric condition associated with polymorphic CGG expansions, POI/POF) [19], offering until recently the only hint of an ovary-related function of *FMR1*.

In elucidating some of this function, we newly defined genotypes, based on entirely different CGG count ranges than those defining neuro-psychiatric risks. Based on a normal range of 26 to 34 (median 30) CGG repeats [21], consistent amongst all races [17], those genotypes determine different ovarian aging patterns and, therefore, variable declines in ovarian reserve [21].

At approximately the median of this normal range lies the distribution peak (29–30 CGG repeats), reported by Fu and associates in the general population [22]. Exactly at median (30 CGG repeats) lies the switching point between positive and negative message and point of maximal translation of the gene product (fragile X mental retardation protein, FMRP), reported by Chen et al. [23].

CGG counts on the two X chromosome alleles define whether a genotype is normal (*norm*), heterozygous (*het*) or homozygous (*hom*). *Norm* women demonstrate distinctively different ovarian aging from *het* and *hom* genotypes [21]. Since ovarian reserve at all ages greatly affects treatment success of infertile patients [24], *FMR1* genotypes should, therefore, demonstrate significant impact on treatment outcomes.

Whether race/ethnicity affects impacts of *FMR1* genotypes on IVF is unknown. How *FMR1* effects differ between races/ethnicities was recently suggested a desirable study subject [25]. *FMR1* genotype differences could represent possible explanations for observed differences in infertility treatment outcomes.

## Methods

### Patients

We investigated 339 consecutive infertility patients, who have been subject of a prior report [26]. Their characteristics are summarized in Table 1: Mean age was 38±5 years; mean BMI 24±5; mean AMH at time of presentation was 1.4±1.7 ng/mL; mean baseline follicle stimulating hormone (FSH) 11.0±6.4 mIU/mL; mean estradiol was 53.1±39.9 ng/mL.

**Table 1:** Patient characteristics.

	Caucasian	African	Asian	Total
	n = 232	n = 59	n = 48	n = 339
Age (years)	38±5	38±5	38±5	38±5
BMI	24±5^1,2^	26±6^1,3^	22±3^2,3^	24±5
AMH (ng/mL)	1.3±1.6	2.2±1.3	1.4±1.3	1.4±1.7
FSH (mIU/mL)	11.0±6.5	11.8±7.5	10.2±4.8	11.0±6.4
Estradiol (ng/mL)	55.7±46.8	50.3±17.5	44.4±17.9	53.1±39.9
*IVF Parameters*				
Oocytes	8±7	7±7	7±6	8±7
Embryos	2±1	2±1	2±1	2±1
Cancelled cycles (%)	10 (4.3)	3 (5.1)	4 (8.3)	17 (5.0)
Pregnancies (%)	60 (25.9)^4^	6 (10.2)^4,5^	14 (29.2)^5^	80 (23.6)
*FMR1* Genotypes (%)				
*norm*	127 (54.7)	31 (52.5)	25 (52.1)	183 (54.0)
*het-norm/high*	39 (16.8)	9 (15.3)	14 (29.2)	62 (18.3)
*het-norm/low*	66 (28.4)	19 (32.2)	9 (18.8)	94 (27.7)

Differences between racial/ethnic groups were not significant except for where noted with P values: ^1^ = 0.001; ^2^  = 0.03; ^3^<0.0001;^ 4, 5^ <0.05.

Patients self-identified in race/ethnicity (or were excluded) as Caucasian (European descendancies but also including Hispanics self-identified as Caucasians); African (black women of African, Caribbean, Hispanic and Afro-American descent); and Asian (mostly Chinese, minorities with Indian or Pakistani heritage and rarely from other Asian countries); Hispanics mostly self-identified with other ethnic groups. Middle-Eastern (of Arab, Druze and Iranian) and Jewish (Ashkenazi and Sephardic) patients were included with Caucasians.

### Laboratory investigations

*FMR1* genotypes were designated based on a normal range of 26–34 (median 30) CGGs [21] and as previously utilized in defining specific patient populations [26]: *norm* was defined by both alleles within range, *het* by one allele outside and *norm/low* or *norm/high*, depending on the abnormal count allele being above or below normal range. Both alleles outside range defined *hom.* As *hom* patients represented only 1–2 percent in all groups, statistical analysis was impossible, and they were excluded.

*FMR1* analyses utilized routine clinical assay systems [21]. Hormone assays were performed in house [21].

Patients were defined as either non-immune or autoimmune based on a previously reported laboratory screen, and considered autoimmune with any one positive test [26]–[28]. This definition consciously selects sensitivity over specificity and biases against findings of statistical associations with presence of autoimmunity. This panel, however, previously has been demonstrated to define risk towards premature ovarian senescence [27], [28] and associations between *FMR1* sub-genotypes, a slim PCO-like phenotype and autoimmunity [26].

### In vitro fertilization (IVF) outcomes

IVF data were extracted from the center's confidential electronic data base. Data in question resulted in manual chart review.

### Statistical analysis

Data are shown as mean ± SD or as raw numbers and percentages. Demographic and biochemical data were analyzed with one way analysis of variance or with chi square. When an analysis of variance was significant, Student Neuman Keuls (SNK) test was used to analyze differences between group means. Differences between groups in proportions of pregnancies established with IVF were analyzed with univariate logistic regression, and were considered significant at P<0.05.

Multivariate logistic regression models of clinical pregnancy after IVF were then used for covariates, such as age and different ovarian reserve parameters, and to examine separate and combined effects of *FMR1* genotypes and race/ethnicity.

### Institutional Review Board

All data utilized in this study were extracted from medical records and/or the center's confidential electronic research data base. Patients, at initial consultation, routinely sign an informed consent, which allows members of the center to use their medical records for research purposes as long as their identity is protected and all medical information remains confidential. These conditions were met for this study and the study, therefore, qualified for expedited review by the center's Institutional Review Board.

## Results

The study group of 339 divided into 232 Caucasian, 59 African and 48 Asian women. Table 1 summarizes patient characteristics. Mean age was 38±5 years and races/ethnicities did not vary. Mean BMI was 24±5 but Asians had the significantly lowest (22±3; p<0.0001 vs African, p = 0.03 vs. Caucasian), followed by Caucasians (24±5; vs. African p = 0.01) and Africans (26±6). AMH, FSH and estradiol did not differ.

Numbers of oocytes, transferred embryos and cycle cancellations also did not differ. In univariate analysis clinical pregnancy rates were, however, in Africans significantly lower than in Caucasians and Asians (both P<0.05). Correcting for cycle cancellations, the difference in clinical pregnancy rates between Africans and Caucasians/Asians was maintained (both P<0.05).

*FMR1* genotypes also did not reach significant differences, though Asians demonstrated a trend towards more frequent *het-norm/high* and Africans towards *het-norm/low* (Table 1).

Logistic regression confirmed *FMR1* genotypes overall as predictive of pregnancy rate (P = 0.046), with most significance through *het-norm/low* (OR 0.44; 95% CI 0.23–0.85; P = 0.014) in comparison to *norm*. *Het-norm/high* did not differ from *norm* in predicting odds of pregnancy (OR 0.73; 95% CI 0.37–1.45; N.S.).

Adjusting for age and BMI increased the overall significance of *FMR1* (P = 0.03) and did not change significance of *het-norm/low* (OR 0.42; 95% CI 0.21–0.83; P = 0.013).

When race/ethnicity was investigated by logistic regression, pregnancy rates differed significantly (P = 0.03). African demonstrated significantly reduced odds of pregnancy compared to Caucasian (OR 0.33, 95% CI 0.13–0.79; P = 0.014). Adjustment for age did not change odds of pregnancy (P = 0.03 for overall race/ethnicity and OR 0.31, 95% CI 0.12–0.79; P = 0.01 for African in comparison to Caucasian). Adding BMI, overall significance was also maintained (P = 0.02) and African race/ethnicity further improved the association (OR 0.27, 0.10–0.70; P = 0.007).

Including in logistic regression *FMR1* genotype, age and BMI, race/ethnicity remained significant (P = 0.03), African continued to have significantly decreased odds of pregnancy compared to Caucasian (OR 0.27, 95% CI 0.10–0.72; P = 0.009), and within the *FMR1* categories *het-norm/low* continued decreased odds of pregnancy compared to *normal* (OR 0.44, 95% CI 0.22–0.89; P = 0.02). Further adjusting this model with the log of individual AMH values (a representation of ovarian function) caused the *FMR1* factor to lose significance but did not significantly change effects of race/ethnicity.

**Figure 1:**
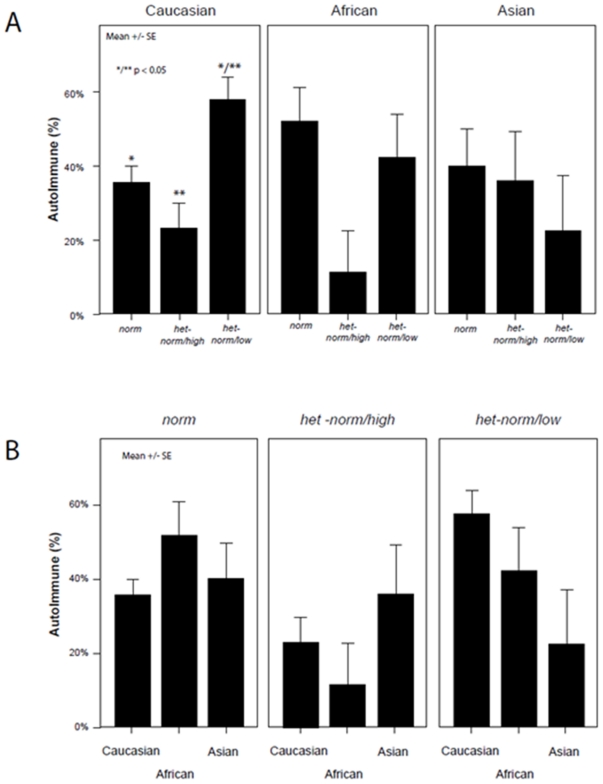
Prevalence of autoimmunity based on race/ethnicity and *FMR1* genotype. Autoimmunity, overall, did not differ amongst the three races/ethnicities. Panel A, however demonstrates differences in prevalence of autoimmunity within races, while Panel B demonstrates the same data stratified by *FMR1* genotype. The interaction between race/ethnicity and *FMR1* genotypes, overall, almost reached significance (P = 0.07), suggesting different *FMR1* effects in the three races/ethnicities. Logistic regression, with race/ethnicity and interaction between *FMR1* genotype and autoimmunity in the model, has 2.5-times the odds of being associated with autoimmune positivity (OR 2.5, 1.34–4.55; P = 0.004).

We previously reported that in this patient population autoimmunity almost reached independent association in predicting pregnancy (P = 0.06) [26]. Figure 1 demonstrates the prevalence of autoimmunity. Differences, overall, did not reach significance. The upper panel (A), however, demonstrates differences within racial/ethnic groups based on *FMR1* genotype. The lower panel (B) offers further clarifications when the same data are shown stratified by race/ethnicity within each *FMR1* genotype.

Autoimmunity was overall most frequent amongst African women (n = 25, 42.4%), followed by Caucasians (n = 92, 39.7%) and least among Asians (n = 17, 35.4%). The interaction between *FMR1* genotype and autoimmunity almost reached significance (P = 0.07), suggesting different *FMR1* effects in races/ethnicities. Association of autoimmunity in *het-norm/low* was most pronounced in Caucasians, followed by Africans and the least in Asians. *Het-norm/high* reduces autoimmunity risk significantly in Caucasians (P<0.05). A similar non-significant trend was observed for Africans. A non-significant increased proportion of individuals with autoimmunity was observed among Asians. Risk towards autoimmunity in all three races/ethnicities appears least affected with *norm FMR1*.

Logistic regression, with race/ethnicity and interaction between *FMR1* genotype and autoimmunity in the model, has 2.5-times the odds of being associated with autoimmune positivity (OR 2.5, 1.34–4.55; P = 0.004).

## Discussion

Race/ethnicity affects treatment outcomes in infertility [1]–[9]. Minority populations, whether of African [1], [4], [7], [10], Asian [1]–[3], [5]–[9] or Hispanic [7] descent, demonstrate lower pregnancy success than Caucasians. Why, has remained unresolved.

Searching for explanations for lower pregnancy rates in Chinese women, we reported more POA/OPOI in Chinese than Caucasian oocyte donors [13]. At the same time CGG trinucleotide repeats in high-normal and intermediate ranges were associated with premature FSH elevations [29], [30]. We observed that 26–34 CGG repeats represent normal in regards to ovarian function [21], while counts below and above denote risk towards POA/OPOI [31].

Distributions of outliers differed among races/ethnicities, with Asians being most, Caucasians least homogenous [17]. Africans (and to lesser degree Caucasians) demonstrated a preponderance for abnormally low and Asians for mostly abnormally high outliers [17]. At that point we, however, were still unaware that newly defined *FMR1* genotypes would be associated with specific ovarian aging patterns, as later demonstrated [21], [26].

Since then, *het-norm/low* has been associated with a PCO-like ovarian phenotype, rapidly depleting follicles (and ovarian reserve) [32], significantly reduced pregnancy chances in IVF and high risk towards autoimmunity (with PCO-like ovarian phenotype 84% likelihood) [26], [33]. In contrast, *het-norm/high*, also associated with risk towards POA/OPOI, reflects lower risk towards poorer IVF pregnancy chances, and, indeed, appears protective against autoimmunity (10% likelihood) [26], [33].

That African women demonstrate decreased IVF pregnancy rates and decreases in pregnancy chance with *het-norm/low* is, therefore, not surprising. These results concur with previously reported observations: African women demonstrate a preponderance of abnormally low count CGG outliers [17], corresponding to *het-norm/low*. Likely missing significance due to small patient numbers, they also demonstrated the highest prevalence of *het-norm/low* (Table 1).

Adjusting for various covariates maintained significant associations for pregnancy outcomes. Further adjusting the model with log of individual AMH values (representative of ovarian reserve) caused the *FMR1* factor, however, to lose significance, while maintaining significance of race/ethnicity. This suggests that *FMR1* effects on pregnancy chances are mostly ovarian, while effects of race/ethnicity are not, and, likely, systemic (i.e., implantation-related). Autoimmunity, of course, therefore comes immediately to mind as potential culprit but remains to be proven.

Based on previously noted associations between *het-norm/low*, autoimmunity and diminished IVF pregnancy chances [26], [33], we expected similar associations here. This study, however, suggests that, at least in regards to autoimmunity, *FMR1* gene effects vary between races/ethnicities. Multiple regression of autoimmune status against race/ethnicity and *FMR1* genotype, and the interaction of the latter two, reached almost significance (p = 0.07; data not shown). Moreover, logistic regression, with race/ethnicity and interaction between *FMR1* genotype and autoimmunity in the model demonstrated 2.5-times the odds of being associated with autoimmunity.

In a different way of assessing results (Figure IB), women with *norm* genotype should in all races/ethnicities represent the approximate average of autoimmune prevalence (all races/ethnicities, indeed, demonstrate similar prevalence). As autoimmunity amongst African women with *het-norm/low* sub-genotype is similar and, therefore, likely approximately normal, Caucasians actually demonstrate excessive autoimmunity with this sub-genotype, and Asians an unusually low prevalence. Asians, indeed, demonstrate the by far lowest prevalence.

As previously reported for women with PCO-like phenotypes [26], [33], *het-norm/high* here also appears protective against autoimmunity. This protective effect, however, extends only to Caucasian and African women and not to Asian patients, who actually demonstrate the highest autoimmune prevalence with *het-norm/high*.

We previously also reported that *het-norm/low* sub-genotype practically halves pregnancy chances in comparison to *norm FMR1* genotype, *het-norm/high* falling in-between [26], [32]. Here presented data, therefore, concur since Africans exhibited lowest pregnancy rates and highest prevalence of sub-genotype *het-norm/low*, while Asians, with lowest prevalence of this sub-genotype, demonstrate highest pregnancy rates.

These observations support *FMR1* contributions to pregnancy outcome differences between races but do not yet allow us to accurately quantify these effects. Indeed, additional association studies on larger patient numbers will be required before *FMR1* trinucleotide repeats will become a clinically useful tool in accurately predicting odds of pregnancy in association with the IVF process. Until such data become available, it appears prudent to consider the *FMR1* genotype of infertility patients. Changes in treatment protocols would, however, appear inappropriate, except in clinical trials, and with appropriate informed consents.

The high IVF pregnancy rate amongst Asians is surprising, contradicts reports of inferior IVF outcomes in Asians [1]–[3], [5]–[8] and our center's own earlier results a number of years ago in egg donors [13]. It, however, correlates well with a recent paper by Huddleston et al., which reported comparable pregnancy rates in Asians and Caucasian egg donors but higher estradiol levels in Asians [9]. Combined, these data may suggest that ovarian function between Asians and Caucasians, indeed, differs but this difference may not be expressed in IVF pregnancy rates until the ovarian reserve is critically diminished at more advanced female age.

Poor pregnancy rates in African women, in contrast, confirm the literature [1], [4], [7], [10]. Why African and Asian patients in this study demonstrate such contradictory findings remains to be determined.

As Table 1 demonstrates, only BMIs differed statistically between groups. While on first glance this difference may appear minor, adding age to the logistic regression marginalized the statistical significance of *FMR1* for pregnancy (P = 0.06) but this significance was fully restored by adding BMI to the regression.

BMI may, therefore, under certain circumstances be more important than generally appreciated. The importance of BMI may relate to PCO-like phenotypes: As noted *het-norm/low* is closely associated with a slim PCO-like phenotype [32]. Significantly higher BMI in Africans would suggest underrepresentation of this phenotype.

It is important to note that the here investigated patient population cannot be considered representative of the typical infertility population seen at average fertility centers. Though mean ages of infertile women reaching IVF are increasing in most developed countries, our center's patients have been exceeding reported age increases, reaching a mean of 39.5 years by 2009. Concomitantly, we witnessed between 2005 and 2009 a switch from over 60% under age 40 to approximately the inverse. In parallel, younger women demonstrated significantly declining ovarian reserve, based on AMH levels (www.centerforhumanreprod.com), when already in 2006 diminished ovarian reserve was recorded in over 50 percent of IVF patients [34].

Specific factors, responsible for unexpectedly good pregnancy outcomes in Asians, can also not be ruled out. One may be utilization of dehydroepiandrosterone (DHEA) supplementation, which greatly improves infertility treatment success in women with diminished ovarian reserve [35]. This beneficial effect, at least theoretically, could disproportionally benefit Asians.

Here reported IVF pregnancy rates in Caucasians and Asians, considering their poor ovarian reserve, are actually beyond expectations, while those of Africans appear more in line with expectations at mean ages of 38 years, FSH of 11.0 mIU/mL and AMH of 1.4 ng/mL. Current treatment interventions with diminished ovarian reserve may, therefore, indeed benefit Caucasians and Asians disproportionally, a hypothesis currently under investigation.

With *FMR1* mapping to Xq27.3 [17], the long arm of the X chromosome appears of increasing importance. For example, in Turner syndrome Xq21 terminal deletions are common, often large, and characterized by primary or secondary amenorrhea [36], [37]. POF/POI demonstrates a 4MB locus exactly at Xq27-q28 [37]. In balanced translocations only Xq23-q27 deletions are associated with POF/POI [37]. The long arm of the X chromosome also contains multiple loci, promoting autoimmunity [37] and, therefore, increasingly looks like a cross roads of ovarian function and autoimmunity [26], [32].

This study confirms that African women experience lower IVF pregnancy rates than Caucasians and Asians. Risk towards lower rates is independently associated with *het-norm/low FMR1*. The association of *FMR1* sub-genotypes and risk/protection for/from autoimmunity suggests that autoimmunity may be associated with lower pregnancy rates in IVF.

Research on the effects of race/ethnicity on reproductive success has to continue, including race/ethnicity – bases assessments of infertility outcomes. Indeed, the reporting of universal treatment outcomes, independent of race/ethnicity, appears counterproductive and, possibly, even misleading.

This study also reaffirms the rapidly evolving importance of *FMR1* in infertility. Initially believed to have only diagnostic importance [29], [30], [21], it now increasingly also assumes prognostic relevance in regards to treatment success [26].

Finally, autoimmunity in reproduction for decades has been highly controversial [38]. Proximity of *FMR1* and autoimmune loci on the long arm of the X chromosome appears not coincidental. Investigations of autoimmune effects in reproduction, therefore, deserve another chance but, probably, require stratification of patient populations based on ovarian function-related *FMR1* genotypes and sub-genotypes.
